# Implied functional crossed cerebello-cerebral diaschisis and interhemispheric compensation during hand grasping more than 20 years after unilateral cerebellar injury in early childhood

**DOI:** 10.1186/s40673-015-0032-0

**Published:** 2015-11-21

**Authors:** Takayuki Nakahachi, Ryouhei Ishii, Leonides Canuet, Masao Iwase

**Affiliations:** Department of Clinical Neuroscience and Psychiatry, Osaka University Graduate School of Medicine, D3 2-2 Yamadaoka, Suita, Osaka 565-0871 Japan; Laboratory of Cognitive and Computational Neuroscience, Center for Biomedical Technology, Madrid Complutense University and Madrid Polytechnic University, Campus Montegancedo, 28223 Pozuelo de Alarcón, Madrid Spain

**Keywords:** Brain injuries, Cerebellar ataxia, Cerebellum, Magnetoencephalography, Near-infrared spectroscopy, Sensorimotor cortex

## Abstract

**Background:**

Crossed cerebello-cerebral diaschisis (CCCD) conventionally refers to decreased resting cerebral activity caused by injury to the contralateral cerebellum. We investigated whether functional activation of a contralesional cerebral cortical region controlling a specific task is reduced during task performance in a patient with a unilateral cerebellar lesion. We also examined functional compensation by the corresponding ipsilesional cerebral cortex. It was hypothesized that dysfunction of the primary sensorimotor cortex (SM1) contralateral to the cerebellar lesion would be detected together with a compensatory increase in neural activity of the ipsilesional SM1. To test these possibilities, we conducted non-invasive functional neuroimaging techniques for bilateral SM1 during hand grasping, a task known to activate predominantly the SM1 contralateral to the grasping hand. Activity in SM1 during hand grasping was measured electrophysiologically by magnetoencephalography and hemodynamically by near-infrared spectroscopy in an adult with mild right hemiataxia associated with a large injury of the right cerebellum due to resection of a tumor in early childhood.

**Results:**

During left hand grasping, increased neural activity was detected predominantly in the right SM1, the typical developmental pattern. In contrast, neural activity increased in the bilateral SM1 with slight right-side dominance during right (ataxic) hand grasping.

**Conclusions:**

This study reported a case that implied functional CCCD and compensatory neural activity in the SM1 during performance of a simple hand motor task in an adult with unilateral cerebellar injury and mild hemiataxia 24 years prior to the study without rehabilitative interventions. This suggests that unilateral cerebellar injuries in early childhood may result in persistent functional abnormalities in the cerebrum into adulthood. Therapeutic treatments that target functional CCCD and interhemispheric compensation might be effective for treating ataxia due to unilateral cerebellar damage.

## Background

Crossed cerebello-cerebral diaschisis (CCCD) is defined as a decrease in cerebral activity contralateral to a cerebellar lesion. It is speculated that CCCD results from dysfunction of contralateral projections from the cerebellum to the cortex via the superior cerebellar peduncles and cerebello-thalamo-cortical pathway [1–7]. Previous single photon emission computed tomography studies on CCCD revealed reduced metabolism and blood flow in the contralesional cerebral cortex during resting state. However, it is unclear whether the loss of a function controlled by a specific contralesional cerebral region can be induced by a cerebellar lesion. Accordingly, in this study we used functional imaging to investigate whether cortical activity of the contralesional primary sensorimotor cortex (SM1) was reduced relative to the normal developmental pattern during simple hand movement in a patient with a unilateral cerebellar lesion and mild hemiataxia.

Numerous functional imaging studies have revealed that hand grasping activates predominantly the SM1 contralateral to the grasping hand [8–14]. We therefore measured bilateral SM1 activity during left and during ataxic right hand grasping using magnetoencephalography (MEG) and near-infrared spectroscopy (NIRS) in an adult patient who had extensive right cerebellar and partial vermal injury due to tumor removal in early childhood. We speculated that the ipsilesional (right) SM1 would be activated predominantly during contralesional (left) hand grasping as expected for a healthy adult, whereas the contralesional (left) SM1 would show relatively less activation during ataxic right hand grasping, possibly with compensatory activity in the ipsilateral (right) SM1.

## Case presentation

The male patient of this study was 30 years old when the final data were acquired. Neurological examination revealed only mild right hemiataxia and disturbance of skilled movements including fine and gross motor skills, e.g. slow movements, difficulties in the development of motor skills, awkward handwriting, poor postural control and difficult articulation. Furthermore, he was found to have constructional apraxia as assessed by an experienced neuropsychologist. This was supported by a poor visuo-spatial information processing and low scores on the block design, object assembly and digit symbol substitution subtests of the Wechsler adult intelligence scale-revised. Scoring on performance IQ was lower by 55 points than that of the verbal IQ. The neuropsychological report suggested that the patient may require more effort for movement initiation and execution and for speech, stemming from his apraxia and lack of confidence in his performance related to cerebellar dysfunction. According to a recent review on cerebellum and apraxia [15], the symptoms of this patient likely meet diagnosis of developmental coordination disorder with mild apraxic agraphia and apraxia of speech.

When the patient was 4 years old, action tremor in the extremities, staggering gait, clumsiness and other signs of delayed motor development became apparent. These abnormalities affected his daily activities and could be noticed by his parents and kindergarten’s teachers. This led his parents to visit pediatricians and neurosurgeons of the community hospital. A cerebellar tumor was found by computed tomography and a subtotal resection was performed, resulting in removal of a broad area of the right cerebellar hemisphere and part of the vermis. Mild right hemiataxia manifested thereafter. In addition, the onset of the disease had changed his dominant hand from right to left according to his mother report. Prior to this study, switching of his dominant hand at 3 years of age was confirmed based on observations of 23 childhood photographs showing use of the right hand in 13/14 photos taken at 1 and 2 years of age compared to only 1/9 photos taken at 3–5 years of age. Furthermore, the Edinburgh handedness inventory [16] conducted at 26 years of age confirmed left-hand dominance. The patient reported the severity of mild right hemiataxia had been constant while left extremity function had been almost intact since 4 years of age.

When he was 6 years old, he experienced sudden severe headache and vomiting caused by hydrocephalus and received a ventriculoperitoneal shunt. At 8 years, he underwent subtotal resection of the residual cerebellar tumor due to relapse. After this surgery, rapid tremor appeared occasionally during isometric contraction of the right fingertips. When he was 18 years old, expansion of the cyst around the tumor was treated surgically and biopsy revealed that the tumor was a pilocytic astrocytoma. At 20 years, he underwent gamma knife radiosurgery (maximal dose: 20 Gy, marginal dose: 12 Gy). Due to relapse and cyst expansion at age 24, gamma knife radiosurgery (maximal dose: 28 Gy, marginal dose: 14 Gy) was repeated together with reoperation of the ventriculoperitoneal shunt, after which he showed stable disease, including throughout the duration of this study.

**Fig. 1:**
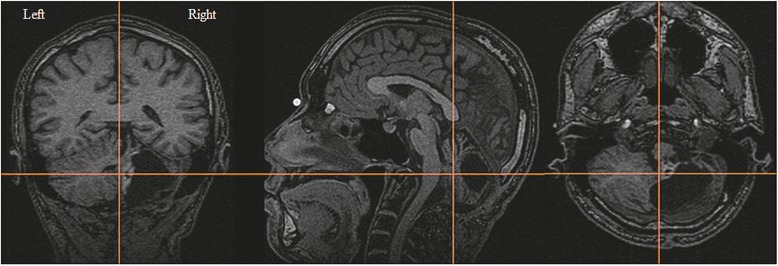
Brain MRI T1-weighted images of the patient. The positions of the coronal, sagittal, and axial images are indicated by the orange lines. A mural nodule of the pilocytic astrocytoma is shown near the crossing point of the orange lines on the coronal and axial images. A cyst associated with the tumor occupies most of right cerebellum

T1-weighted anatomic MRI images acquired prior to this study are shown in Fig. 1. The residual pilocytic astrocytoma can be seen at the cerebellar vermis adjacent to the medulla oblongata, and most of the right cerebellar hemisphere is filled by the cyst associated with the tumor. The patient had not received specific interventions or rehabilitation for the mild right hemiataxia prior to the study. Therefore, this case allowed for investigation of the natural neurodevelopmental changes over the 24 years since unilateral injury to the cerebellum.

## Materials and methods

Cortical activity in SM1 was assessed by MEG and NIRS at 28 and 30 years of age, respectively, as a part of research projects on neurodevelopmental disorders. These experimental procedures were approved by the Ethics Committee of Osaka University Graduate School of Medicine and conformed to all policies and principles contained in the Declaration of Helsinki.

MEG measures the magnetic fields associated with electrophysiological activity. Event-related desynchronization in the beta oscillation (beta ERD), a robust electrophysiological correlate of movement, was measured by MEG during left and right hand grasping using a 64-channel whole head magnetometer (NeuroSQUID Model 100, CTF Systems Inc., Canada). In healthy adults, the beta ERD is detected by MEG dominantly in the contralateral SM1 during hand grasping [8, 11, 13]. During data collection, the patient sat on a chair in a magnetically shielded room. The hand grasping task comprised of eight trials, each of 15 s duration, equally divided into three stages: (1) pre-movement, (2) movement, and (3) post-movement. During the pre/post-movement stages, the patient was in a resting state, sitting still with his eyes open. During the movement stage, he was asked to perform the task—self-paced repeated hand grasping of a rubber ball with the right or left hand—and not perform similar movements except for the task. MEG signals were digitized at 625 Hz and filtered using a combined 60 Hz notch filter and 200 Hz low-pass filter. For each trial, the beginning of the movement stage (5-s duration) was considered time zero (t = 0). The event-related time-frequency spectrum of the beta ERD was calculated by subtracting the signal of a 4-s epoch (t = −4.5 to −0.5 s) during the pre-movement stage (resting period) from that of the 0.5–4.5 s time-window in the movement stage (task period). Artifact rejection was performed off-line. These experimental procedures were based on Honaga et al. [14]. The spatial distributions of the oscillatory power changes in specific frequency bands were estimated from the raw MEG signals using synthetic aperture magnetometry (SAM), a spatial filtering technique based on the nonlinear constrained minimum-variance beamformer algorithm. In SAM analysis, after filtering into a frequency band, each voxel in the region of interest is linked to the sensor array using a spatial filter optimized for each voxel so that neural activity at the target voxel is emphasized and covarying signals from other voxels are attenuated. Using a spatial filter related to each target voxel, a current source density value was derived for each voxel as a function of time from the MEG signals. Recorded signals were divided into the first state (resting period) and the second state (task period), and the total power in each frequency band was estimated for both states. The difference between these two spectral power values was analyzed using voxel-to-voxel comparison by Student’s *t* test. The distribution of *t* values was fused with the subject’s MR image to generate a SAM statistical image, which was examined for regions of statistically significant power changes in a specific frequency band. The recorded signals were filtered into 4–8, 8–15, and 15–30 Hz bands and subjected to SAM analysis with 5 mm voxel resolution. Details of SAM analysis can be found in Taniguchi et al. and Ishii et al. [8, 17].

**Fig. 2:**
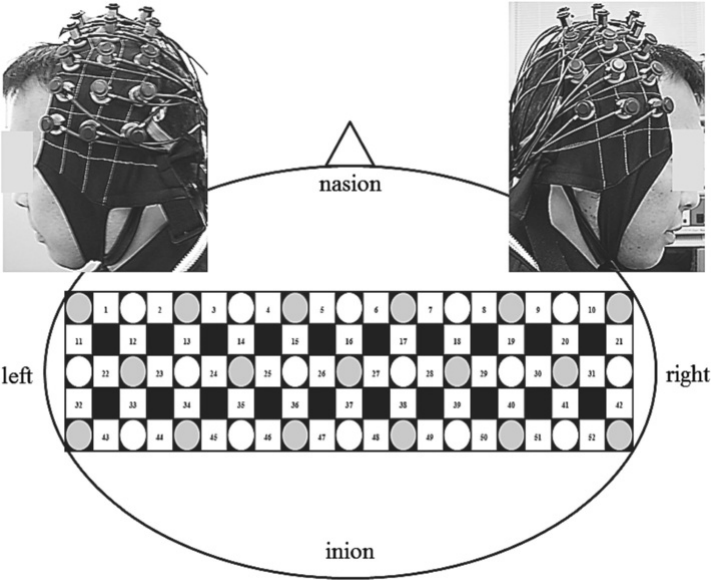
Location of optical diodes and measurement points (channels) on the probe for near-infrared spectroscopy. Channels 1–52 are depicted as white squares between laser diodes (emitters, gray circles) and photodiodes (detectors, white circles)

Hemodynamic responses, which reflect neural activity due to neurovascular coupling [18, 19], were measured in SM1 and adjacent cortical areas during left and right hand grasping by NIRS. Like the MEG signal, NIRS signals in healthy adults are concentrated in the contralateral SM1 during hand grasping [9, 10, 12]. Near-infrared light can penetrate deeply into tissues, where it is differentially absorbed by hemoglobin depending on its oxygenation state and the optical path length in the tissue (as expressed by the modified Beer-Lambert law). Thus, NIRS can reveal cortical hemodynamic responses 2–3 cm below the skin surface by measuring the relative changes in the concentrations of oxygenated hemoglobin ([oxy-Hb]), deoxygenated hemoglobin ([deoxy-Hb]), and total hemoglobin ([total-Hb]). We used a multichannel NIRS with 52 measurement channels (ETG-4000; Hitachi Medical Corporation, Tokyo, Japan). The probe of the ETG-4000 can measure an area approximately 6 cm × 30 cm. The central optical diode was positioned at Cz of the international 10/20 system for electroencephalography to cover the surfaces of both right and left SM1 (Fig. 2). During data collection, the patient sat on a chair in a silent room. A baseline task (30 s), the hand grasping task (90 s), and the baseline task (60 s) were executed in succession using the right or left hand. In the baseline task, the patient was instructed to stare blankly at the front white wall. Relative change in Hb (Δ[Hb]) is expressed as the product of concentration and optical path length (in mM∙mm). In this study, we focused on Δ[oxy-Hb] because it is reported to be most sensitive to changes in regional cerebral blood volume, while the direction of Δ[deoxy-Hb] is determined by variation in both venous blood oxygenation and volume [18]. The ETG-4000 analysis software was used in the “integral mode”. In this configuration, mean Δ[oxy-Hb] during the baseline task 10 s just before starting the hand grasping task or 10 s before finishing the latter baseline task were corrected to 0 mM∙mm by linear fitting. The hand grasping task period itself was set at 90 s and the recovery period from the end of the hand grasping task period until the baseline stabilization was set at 50 s. To smooth out short-term motion artifacts, we employed the moving average method with a 5-s window. The Δ[oxy-Hb] at each channel was measured every 0.1 s during the hand grasping task, and mean Δ[oxy-Hb] values were compiled into a color-coded spatial map. To determine cortical laterality, mean Δ[oxy-Hb] values during the hand grasping task were compared between 25 symmetrical positions using the paired two-tailed Student’s *t*-test, with *p* = 0.05 set as the significance threshold. Next, to delineate differences in cortical activity between the left and right hand grasping tasks, we arranged mean Δ[oxy-Hb] values from all 52 channels in descending order, and investigated channels with mean Δ[oxy-Hb] values more than ± 2 standard deviations from the grand average of all 104 mean values (52 for right hand grasping and 52 for left hand grasping). These experimental procedures were based on Nakahachi et al. [20, 21].

## Results

**Fig. 3:**
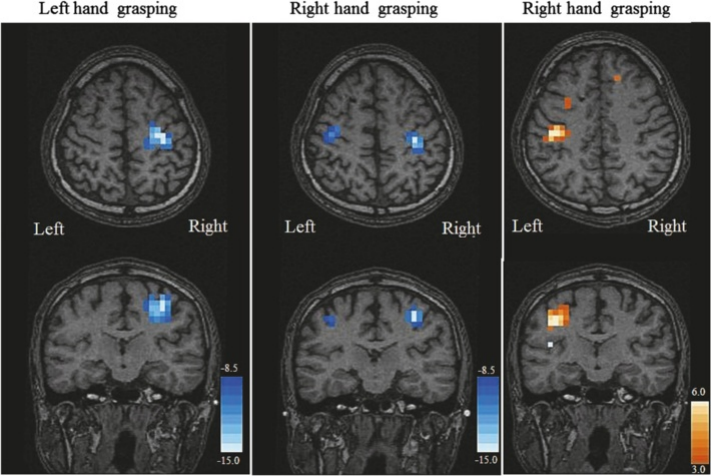
Statistical *t*-maps of event-related desynchronization (ERD) in the beta band (the left two columns) and event-related synchronization (ERS) in the theta band (right column) with significant power changes during the hand grasping task as measured by magnetoencephalography with SAM analysis. The *t*-maps are projected onto T1-weighted MRI images of the patient. The *t* value of each voxel is displayed by a color scale, with decreased power displayed in blue to white and increased in orange to white. During the left hand grasping task, beta ERD was localized in the right primary sensorimotor cortex (SM1), whereas during the right hand grasping task, beta ERD was located in the bilateral SM1 with slight right-side dominance. In addition, theta ERS arose in the left SM1 only during right hand grasping

Analysis of MEG signals revealed that during the left hand grasping task, the beta ERD was localized to the right SM1 as expected, whereas during the right hand grasping task, the beta ERD was located in the bilateral SM1 with right-side dominance (Fig. 3). The left SM1 exhibited a lower beta ERD than the right SM1 during right hand grasping (i.e., right dominance). In addition, there was a substantial increase in power of the theta band event-related synchronization (theta ERS) in the left SM1 during right hand grasping, a response not observed in the contralateral (right) SM1 during left or right hand grasping. Thus, this appears to be a response unique to the cortical source related to the ataxic movement of the right hand. The SM1 area with peak changes corresponded to the motor hand area of the precentral knob [22].

**Fig. 4:**
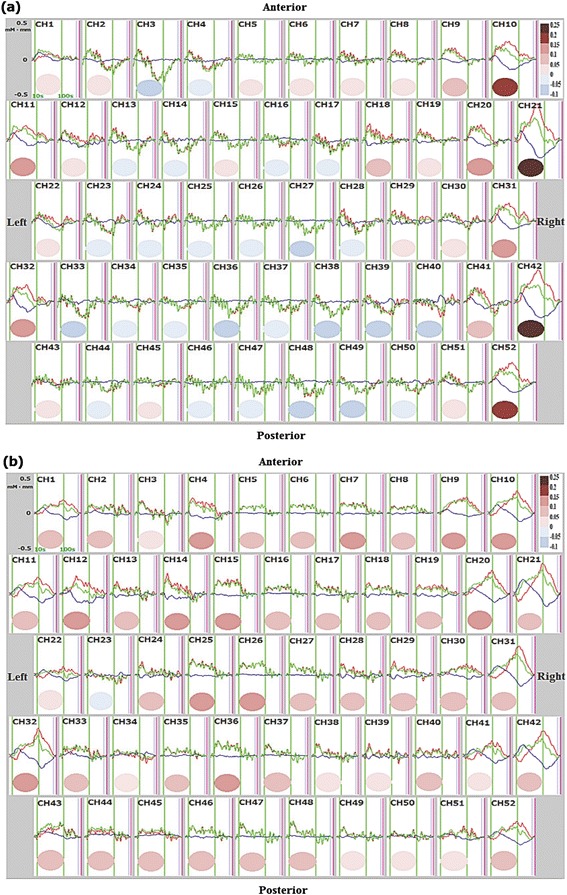
Grand average waveforms of Δ[oxy-Hb] (red), Δ[deoxy-Hb] (blue) and Δ[total-Hb] (green) during left hand grasping (**a**) and right hand grasping (**b**). Individual waveforms are displayed on schema of the probe configuration for near-infrared spectroscopy. For each channel, the *x*-axis denotes time from 0 to 160 s and the *y*-axis denotes activation between −0.5 and 0.5 mM∙mm. The hand grasping task period is marked by two vertical green lines at 10 and 100 s. The mean value of Δ[oxy-Hb] during the hand grasping task for each channel is color-coded in an ellipse according to the color scale at right. During the left hand grasping task, channels showing markedly elevated Δ[oxy-Hb] were located in the right hemisphere, while many channels showing decreased Δ[oxy-Hb] were distributed in the left hemisphere (**a**). During the right hand grasping task, channels showing moderate increases Δ[oxy-Hb] were distributed diffusely across the bilateral hemispheres (**b**). CH, channel

**Fig 5:**
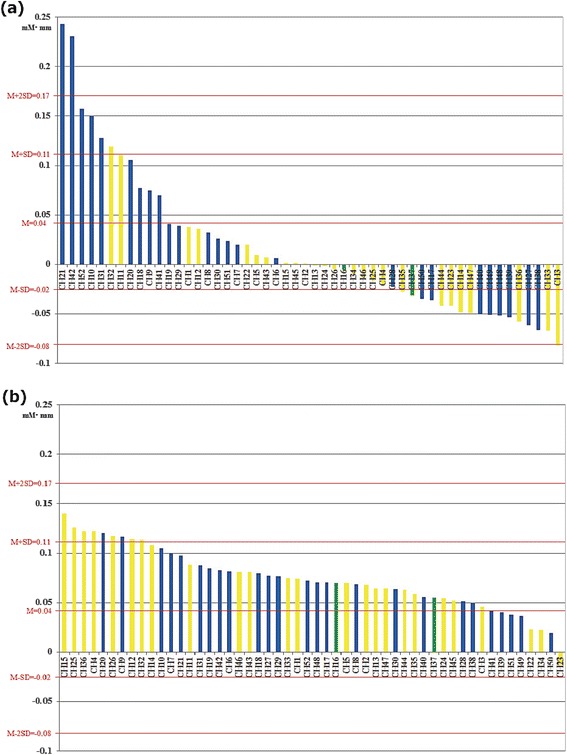
Distribution of mean Δ[oxy-Hb] during the left hand grasping task (**a**) and the right hand grasping task (**b**) in all channels. The *x*-axis denotes channels and the *y*-axis denotes activation between −0.1 and 0.25 mM∙mm. Significance thresholds and average are represented by horizontal red lines. The colors of the vertical bars indicate location of channels: yellow is left, blue is right, green is middle position in the probe. While two significantly activated channels (> M + 2SD) in the right hemisphere and one significantly inhibited channel (< M-2SD) in the left hemisphere were found during the left hand grasping task (**a**), most channels showed moderate activity with no significance during the right hand grasping task (**b**). CH, channel; M, average of mean Δ[oxy-Hb] across all channels for left and right hand grasping; SD, standard deviation of mean Δ[oxy-Hb] across all channels for left and right hand grasping

Analysis of NIRS signals revealed substantial differences in the cortical distribution of mean Δ[oxy-Hb] values between the left and right hand grasping tasks (Figs. 4 and 5). To determine the degree of cortical laterality, the mean Δ[oxy-Hb] values from corresponding right and left hemispheric channels were compared for the left and right hand grasping tasks. During the left hand grasping task, the right hemisphere was significantly more activated (greater Δ[oxy-Hb]) than the left (*d.f.* = 24, *t* = −3.395, *p* = 0.002), consistent with the MEG results. However, there was no significant difference between hemispheres during the right hand grasping task (*d.f.* = 24, *t* = 0.734, *p* = 0.470). Next, the significance thresholds defined by ± 2 standard deviations from the grand average of mean Δ[oxy-Hb] values (0.045 ± 0.126 mM∙mm) were superimposed on the distribution diagrams of all mean Δ[oxy-Hb] for the left and right hand grasping tasks. This analysis indicated that while there were no significant channels during the right hand grasping task, three channels with significant Δ[oxy-Hb] values were seen during the left hand grasping task: significant increases in two right hemisphere channels, and a significant decrease in a left hemisphere channel (Fig. 5).

## Discussion

An adult with mild right hemiataxia due to right cerebellar injury in early childhood presented with decreased neural activity in the contralesional (left) SM1 compared to a typical developmental pattern during right hand grasping, accompanied by enhanced ipsilesional (right) SM1 activity. These neurodevelopment changes likely reflect CCCD or a similar phenomenon together with neural compensation.

Simple one-handed movements are controlled mainly by the contralateral SM1 hand area with critical regulatory input from the cerebellum contralateral to the SM1 [23, 24]. In MEG, the beta ERD appeared in the right SM1 during left hand grasping, the typical response of healthy adults [8, 11, 13], but in the bilateral SM1 with slight right-side dominance during ataxic right hand grasping. This beta ERD pattern is consistent with that reported in patients with gliomas around the central sulcus or with occlusive disease in the arteries providing blood supply to the cerebrum during contralesional hand grasping [11, 13], presumably due to compensatory cortical activity by the contralesional (ipsilateral) SM1. It has been suggested that the hand is innervated by motor pathways from both the contralateral and ipsilateral SM1, and that by 10 years of age, the motor signals from the ipsilateral SM1 are inhibited through transcallosal fibers from the contralateral SM1, thereby establishing the contralateral-dominant pattern observed in typical development [25, 26]. Oshino et al. [13] speculated that a defect of this inhibitory function by the ipsilesional (contralateral) SM1 led to a dominant ipsilateral beta ERD distribution during contralesional hand grasping in a patient with a cerebral hemispheric lesion.

During ataxic right hand grasping, the left SM1 exhibited theta ERS, which is not a usual movement-related oscillation (Fig. 3). While we know of no reports on movement-related theta ERS, MEG measurements of patients with stroke affecting the middle cerebral artery territory have shown enhanced power of low-frequency oscillations, including the theta band, and reduced power of high-frequency oscillations in the perilesional rolandic area of the affected hemisphere (and to a lesser extent in the homotopic unaffected hemisphere i.e., transhemispheric diaschisis) during resting state [27–29]. Laaksonen et al. [29] speculated that such low frequency oscillations in the impaired cortex reflect the allocation of resources for recovery or compensation, but this suggestion remains speculative and future studies are needed to evaluate the functional significance of increased low-frequency oscillations during stroke recovery or in CCCD.

Consistent with MEG measurements, NIRS revealed a contralateral (right) dominant SM1 activation pattern during left hand grasping typical of developmentally normal adults [9, 12], but aberrant bilateral SM1 activation during ataxic right hand grasping, with no significant difference in activity (mean Δ[oxy-Hb] signal) between hemispheres. This pattern was also observed in the SM1 of patients with cerebral stroke during contralesional hand grasping [12], again suggesting that the ectopic beta ERD and blood flow responses reflect compensatory increases in neural activation.

The spatial distribution of hemodynamic changes measured by NIRS revealed a clear dichotomy in metabolic response between the contralateral and ipsilateral SM1 during left hand grasping. This was characterized by an increased blood flow in the right SM1 concomitant with below-baseline activity in the left, whereas the signal in the ipsilateral cortex was reversed relative to the normal response (i.e., increased Δ[oxy-Hb]) in the right SM1 during ataxic right hand grasping (Figs. 4 and 5). Such a diffuse cortical activity pattern, like the bilateral beta ERD, has been reported in brain-injured patients, suggesting that dysfunction of the left SM1 caused by CCCD may induce reorganization and vicariation by the contralateral homologous cortex through neural plasticity and/or failure of interhemispheric inhibition to maintain the normal region-specific activity pattern [26, 30–32].

The MEG and NIRS measurements showed qualitatively similar results, with the exception of slight right-side dominance on MEG compared to nearly equal bilateral cortical activation on NIRS during ataxic right hand grasping. This discrepancy may be attributed to the differences in region of interest, spatial resolution, index for activation, and/or thresholds used in statistical analyses between the two modalities.

In this study, we detected a phenomenon similar to conventional (resting state) CCCD during region-specific cortical activation, which may be termed “functional CCCD” or functional diaschisis (for a review, see [33]). Many studies on patients with unilateral cerebellar injuries have reported an association with contralateral cerebral hemispheric dysfunction (even if “crossed cerebello-cerebral diaschisis” was not used to describe the phenomena), including reduced verbal and visuo-spatial abilities caused by right and left cerebellar injuries, respectively [4, 5, 7, 23]. However, these studies did not directly measure cortical activity during tasks, just the behavioral results, or measured reduced neural metabolism or activity only during the resting state. Moreover, most of the tasks used in these studies are not associated with such spatially restricted and predictable activity patterns as in simple hand movements. A previous case report did, however, demonstrate crossed cerebro-cerebellar diaschisis (CCD, cerebellar dysfunction associated with contralateral forebrain injury) during simple hand motor tasks with confirmed laterality by positron emission tomography (PET). Di Piero et al. [34] demonstrated right CCD and right hand clumsiness during resting state 10 days after a lacunar hemorrhage in the left globus pallidus. After 1 month, right hand clumsiness and laterality of blood flow in cerebellar hemispheres during the resting state recovered. However, blood flow remained higher in the left cerebellar hemisphere than in the right hemisphere during a finger opposition task whether performed by the right or left hand. In other words, this patient exhibited “functional CCD” in the right cerebellum and compensatory neural activity in the left cerebellum. Although properly a case of CCD not CCCD, these results are in accord with those of our study, which showed decreased activity in the area of diaschisis mediated by cerebrocerebellar pathways and increased activity in the contralateral homologue by neural compensation during a simple hand motor task.

We can only speculate on the neurodevelopmental processes that account for diaschisis and neural compensation in our patient because these abnormalities were detected 24 and 26 years after cerebellar injury in early childhood, which to our knowledge is the longest period after the original injury in studies on CCCD or CCD [35]. Increased activity of the contralesional motor pathway has been reported frequently during recovery in adult patients with cerebral stroke (for a review, see [26]). In normal infants, hand movement is associated with activity in the bilateral corticospinal tract, but as the brain matures, the tract from the ipsilateral SM1 is inhibited. This dual activity is presumably why pediatric patients with cerebral stroke demonstrate such reliable and rapid neural compensation by the contralesional SM1 [25, 26]. Many studies have demonstrated CCCD and neural compensation by the non-affected SM1 within a month after injury [1–3, 12, 26, 30]. Therefore, it is likely that functional CCCD and neural compensation in our patient occurred in early childhood, given the findings of previous studies and the switch in handedness at 3 years of age. Furthermore, the lack of any change in subjective severity suggests little effective compensation over the next two decades. It is reported that patients with cerebral stroke who recovered by compensational motor pathway in the non-affected hemisphere show worse motor function than those who recovered by perilesional reorganization in the affected hemisphere which potentially occurs due to early and intensive appropriate rehabilitative interventions [26, 32]. Since the patient of this study had not received such treatment, it would partly explain the maintained neural compensation by the non-affected hemisphere.

The significance of this case study for rehabilitation medicine is that unilateral cerebellar injury in early childhood without specific rehabilitative interventions can result in sustained ipsilesional mild hemiataxia, functional CCCD in the contralesional SM1, and compensatory neural activity in the ipsilesional SM1, which may persist into adulthood. The patient in this study performs most activities of daily living using his left limbs and reports that he would become remarkably disabled if left limb function was like that of his right limbs. Protracted compensatory neural activity by the SM1 in the non-affected hemisphere is generally considered to be the cause of poor motor outcome, and rehabilitative interventions that inhibit the compensatory activity are recommended, such as transcranial magnetic stimulation during the early stage after injury to facilitate the reallocation of activity back to the affected SM1 [12, 26, 32]. In the future, we aim to investigate changes in the cerebello-thalamo-cortical pathway and cortico-spinal tract, such as Wallerian degeneration, which has potential for estimating the possibility of recovery of innervation for hand motor function from the ipsilateral dominant pattern to the contralateral dominant pattern [26, 30, 32, 36, 37], using diffusion tensor imaging. The data from such an investigation could support the application of individualized neurorehabilitative interventions for recovery of hand motor function.

This study has several limitations. First, we included no sex- or age-matched healthy controls for comparison of cortical activation. Nevertheless, we used a task that is thought to predominantly engage the contralateral SM1 hand motor area in healthy subjects, as demonstrated by MEG and NIRS studies [8, 9, 11–13]. Furthermore, data acquired during left hand grasping served as an internal control. Second, we did not demonstrate conventional CCCD, which occurs in the resting state. However, in a previous PET study on CCD, functional diaschisis was detected during a simple hand motor task even though CCD during the resting state had been resolved [34]. Therefore, conventional CCCD and “functional CCCD” may also show independence at the expression or mechanistic levels.

## Conclusions

As far as we know, this is the first study to demonstrate functional CCCD and compensatory neural activity in the cerebral cortex during task performance by functional neuroimaging. The results suggest that unilateral cerebellar injuries in early childhood may result in sustained functional CCCD and interhemispheric compensation in the SM1 persisting into adulthood. Thus, long-term therapeutic treatments, including rehabilitation, that take cerebral dysfunction into consideration may be required even for a unilateral cerebellar focal lesion. In the last two decades, it has been found that the cerebellum contributes to cognition as well as to motor control [5–7, 23, 25, 38]. Therefore, future studies should examine whether functional CCCD is found in supratentorial cognitive areas, especially regions of the prefrontal cortex important for executive control, in patients showing psychiatric symptoms following cerebellar injury.

## Consent

The patient has given written informed consent for the case report and images to be published.

## Abbreviations

beta ERD: event-related desynchronization in the beta band
CCCD: crossed cerebello-cerebral diaschisis
CCD: crossed cerebro-cerebellar diaschisis
deoxy-Hb: deoxygenated hemoglobin
MEG: magnetoencephalography
NIRS: near-infrared spectroscopy
oxy-Hb: oxygenated hemoglobin
SM1: primary sensorimotor cortex
theta ERS: event-related synchronization in the theta band
